# A Step-by-Step Approach to Improve Clinical Translation of Liposome-Based Nanomaterials, a Focus on Innate Immune and Inflammatory Responses

**DOI:** 10.3390/ijms22020820

**Published:** 2021-01-15

**Authors:** Giacomo Della Camera, Dorelia Lipsa, Dora Mehn, Paola Italiani, Diana Boraschi, Sabrina Gioria

**Affiliations:** 1 European Commission, Joint Research Centre (JRC), 21027 Ispra, Italy; giacomo.d.c.88@gmail.com (G.D.C.); dorelia.lipsa@ec.europa.eu (D.L.); dora.mehn@ec.europa.eu (D.M.); 2 Institute of Biochemistry and Cell Biology (IBBC), National Research Council (CNR), 80131 Naples, Italy; paola.italiani@ibbc.cnr.it (P.I.); diana.boraschi@gmail.com (D.B.)

**Keywords:** nanomaterial, liposome, safety assessment, nanomedicine, particle size distribution, physicochemical characterization, Limulus Amoebocyte Lisate (LAL), endotoxin, inflammation, interleukin, cytokines

## Abstract

This study aims to provide guidelines to design and perform a robust and reliable physical-chemical characterization of liposome-based nanomaterials, and to support method development with a specific focus on their inflammation-inducing potential. Out of eight differently functionalized liposomes selected as “case-studies”, three passed the physical-chemical characterization (in terms of size-distribution, homogeneity and stability) and the screening for bacterial contamination (sterility and apyrogenicity). Although all three were non-cytotoxic when tested in vitro, they showed a different capacity to activate human blood cells. HSPC/CHOL-coated liposomes elicited the production of several inflammation-related cytokines, while DPPC/CHOL- or DSPC/CHOL-functionalized liposomes did not. This work underlines the need for accurate characterization at multiple levels and the use of reliable in vitro methods, in order to obtain a realistic assessment of liposome-induced human inflammatory response, as a fundamental requirement of nanosafety regulations.

## 1. Introduction

In recent years, the global liposome drug market has rapidly expanded, mainly due to the suitability of liposomes as safe and effective delivery systems [1]. Indeed, the key drivers for pharmaceutical companies to invest in this product class include advantages such as improvement and control over pharmacokinetics and pharmacodynamics, decreased toxicity, and enhanced activity of drugs, leading to the approval by the USA Food and Drug Administration (FDA) of a number of liposomal products (such as ambisome, myocet, doxil, depoCyt) [2,3,4]. It is nevertheless important to take into account possible safety concerns with the use of liposomes, in particular the potential health risks linked to their intravenous administration. In fact, it is well known that some of the nanomaterial (NM) properties can greatly affect their biocompatibility while in the blood stream [5], and that particular attention should be given to the innate immune system, the first-line defensive mechanism able to identify and destroy potentially dangerous foreign agents, including NM entities [6].

Innate immunity encompasses a number of effector cells (e.g., neutrophils, monocytes, macrophages, natural killer cells) and soluble factors (e.g., complement, cytokines, chemokines) that contribute to a rapid defensive inflammatory reaction that resolves completely once the potential danger is eliminated. However, if not properly controlled, the innate/inflammatory reaction may lead to tissue damage and chronic inflammatory or autoimmune pathologies [7,8]. Considering the likelihood of interaction of nanomedicines with immune cells and factors present in the bloodstream and in the target tissues, their potential immunomodulatory effects (immunostimulation or immunosuppression, with a consequent impact on human health) deserve particular attention in the regulatory risk-benefit assessment.

From a regulatory perspective, immunotoxic effects are not properly examined in the current immunotoxicological studies for human pharmaceuticals (ICH-S8) testing guidelines [9] and, despite the need for standardized approaches, currently there are no regulatory guidelines for immunotoxicity evaluation that are specific for nanomedicines [10]. In this regard, the National Cancer Institute-Nanotechnology Characterization Laboratory (USNCL) and the European Union Nanomedicine Characterization Laboratory (EUNCL), working alongside the US Food and Drug Administration (FDA) and the European Medicinal Agency (EMA), have validated protocols for assessing in vitro the capacity of NM to induce cytokine release, which have the advantage of avoiding animal experimentation and overcoming the limitations that animal models have in predicting human responses to challenges [11,12,13,14].

Liposomes have been used for many decades as efficient carriers for delivering drugs to phagocytic cells (monocytes, macrophages) [15,16,17] or as carriers/adjuvants for improving vaccine efficacy [18], and their toxic/inflammatory effects were evaluated in parallel. From the safety point of view, regulatory requirements call for an accurate and reliable evaluation of the potential detrimental effects (toxicity, induction of inflammatory reactions) of liposome formulations for clinical use (i.e., already loaded with the drug cargo under investigation) [19,20] and upon intravenous administration. These data are however mostly incomplete, without an appropriate comparison between empty and cargo-loaded liposomes, thereby missing the putative intrinsic toxic/inflammatory effects of empty liposomes, information that would help in selecting the most suitable/less toxic vehicles to be used in clinical formulations.

In addition, although it might not be obvious, the USNCL and EUNCL experience reports several cases of liposome-based product developers failing in properly characterizing the material in its early phase. Most common pitfalls are related to homogeneity, batch-to-batch variability, as well as failure to consider the potential bacterial/endotoxin contamination or the toxicity of the material under development. Failing in doing so has led, in most cases, to product development road-block after years of investment and research. This work was then conceived for sharing the know-how on how to perform an accurate characterization of liposome-based NM. It is intended to provide a complete overview to students who are approaching this field for the first time as well as to more skilled developers who might fail in considering some of the aspects of the characterization process.

For this scope, we applied a multi-tiered approach to eight commercially available liposomes for drug loading, which have to be intended as a “model”. This will contribute to foster research by providing a rational procedure, in agreement with regulatory requirements, and to support a correct assessment of liposome safety from the early stage of their pre-clinical development, which is fundamental for a successful transition into clinical development.

## 2. Results

**Figure 1:**
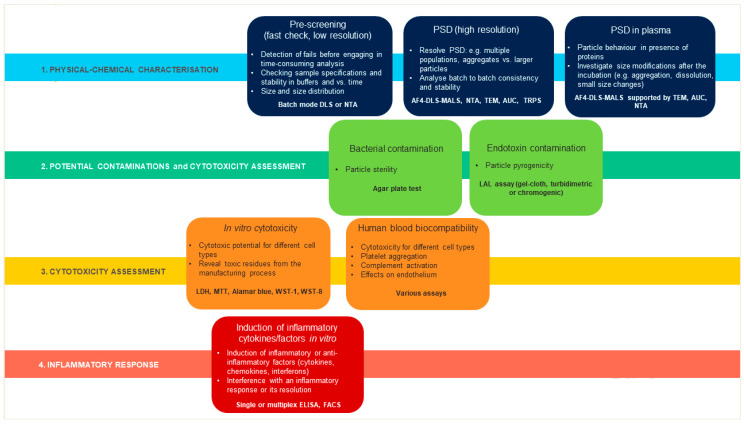
Each liposome was characterized for its physical-chemical properties and for potential contamination by bacteria and bacterial toxins, and eventually examined for toxicity on human cells and capacity to induce an inflammatory response. PSD, particle size distribution.

Eight differently functionalized commercially available liposomes for drug-loading purposes were selected as “case-studies”. The multi-step characterization applied is illustrated in Figure 1.

### 2.1. Physical-Chemical Characterization of Liposomes

UV-Vis spectra were obtained for all the liposomes used in this study (see list in Table 1). Results show that there are no specific peaks of absorption (Figure S1, Supplementary Materials).

**Figure 2:**
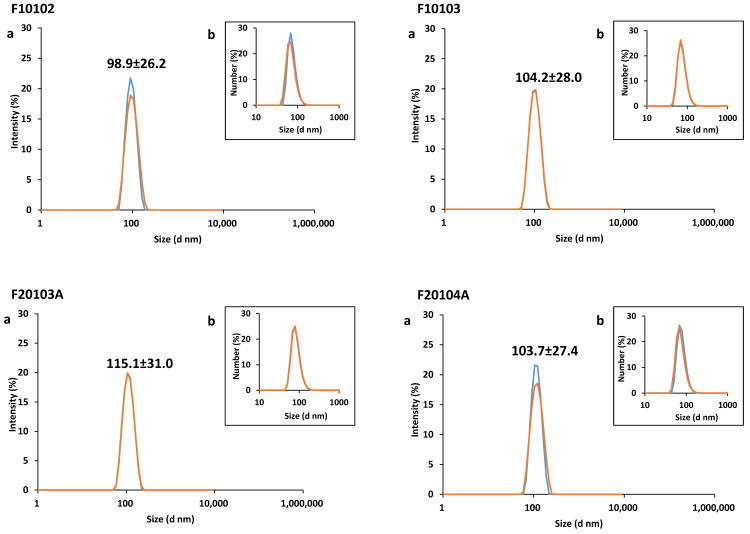

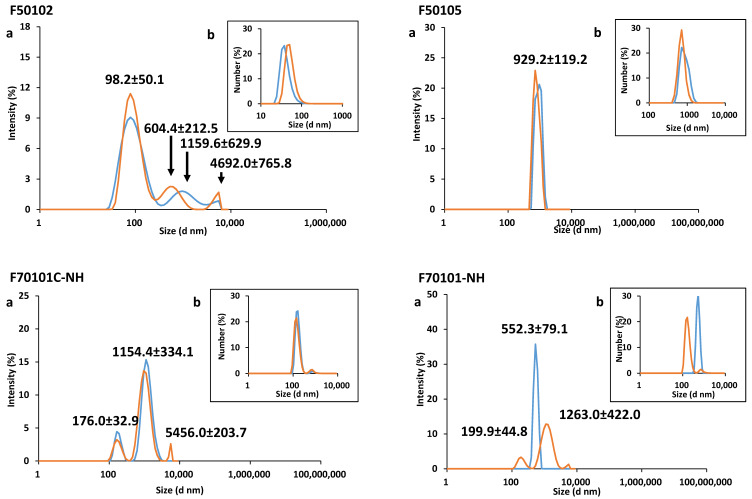
Batch-mode DLS data reported (a) as intensity-based size distribution, and (b) as number-based size distribution. Scheme 25 and 50 µg/mL, (red and blue lines, respectively) were prepared in PBS. Average of three measurements are shown. The PDI of all liposomes is reported in Table 2.

Batch-mode Dynamic Light Scattering (DLS) was performed as preliminary screening to evaluate the particle size distribution (PSD) and the sample monodispersity. Data are summarized in Figure 2 and Table 2. Batch-mode DLS was repeated in time during the whole study to monitor the liposomes’ stability. They proved to be stable for over 6 months (data not shown).

Liposomes F10102, F10103, F20103A, and F20104A resulted in being monodispersed, showing a single intensity peak with a PSD value of 98.9 nm (±26.2), 104.2 nm (±28.0), 115.1 nm (±31.0), and 103.7 nm (±27.4), respectively. A single peak can also be observed when data are expressed in a number-based size distribution (Figure 2). Polydispersity index values are reported in Table 2, and are in accordance with the information provided by the supplier.

A monomodal distribution was registered for liposome F50105, however a very large hydrodynamic diameter of 929.2 nm (±119.28) was observed, with a polydispersity index (PDI) value of 0.49, indicating aggregation of the sample.

Liposomes F50102, F70101-NH, and F70101C-NH showed multimodal intensity-based distributions, with PDI values higher than 0.3. Transformation to number-based distributions provided monomodal curves also for some of these materials, illustrating the risk of a misinterpretation of size distribution data when only shown as number-weighted results.

The liposome zeta-potential and mobility were measured by electrophoretic light scattering (ELS). Data are reported in Table 2 as average values of three measurements. All liposomes were negatively charged, except for F50102, which displayed a charge of +4.1 (±0.39) mV and a mobility of +0.3 (±0.03) µm cm/Vs.

### 2.2. AF4-DLS Analysis

**Figure 3:**
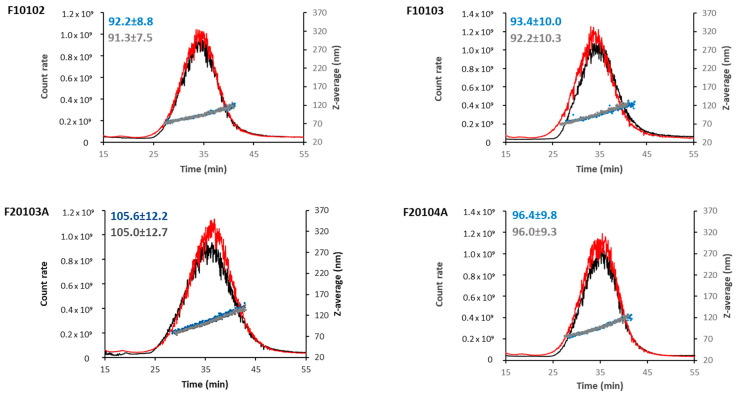
AF4-DLS measurements of selected liposomes in PBS (black lines and blue dots) or PBS supplemented with 10% human serum (red lines and grey dots). The elugram of flow mode DLS (scattered intensity and size vs. elution time) are reported for one representative measurement for each selected liposome and condition. Scattering intensity peaks (lines) and hydrodynamic diameters (dots) by DLS are shown. Hydrodynamic diameter values, measured in PBS or in serum, are reported (blue and grey respectively).

To overcome the batch-mode DLS limitations in the analysis of polydispersed suspensions, liposomes were also tested by Asymmetric Flow Field Flow Fractionation coupled with DLS (AF4-DLS). Data are reported in Figure 3 and Table 2 and expressed as a hydrodynamic diameter (D_h_). Out of the eight liposomes under investigation, only F10102, F10103, F20103, and F20104A were successfully analyzed by AF4-DLS. It was not possible to collect data for the other liposomes although several experimental conditions have been tested.

As shown in Figure 3, at similar elution times (30–35 min), liposomes F10102, F10103, F20103A, and F20104A presented an elution peak with average hydrodynamic diameters of 92.2, 93.4, 105.6, and 96.4 nm, respectively.

It was not possible to acquire data of liposomes F50102, F50105, F70101-NH, and F70101C-NH, likely because of their zeta-potential close to zero, suggesting instability in the solution. Materials with a neutral charge might have interacted too strongly with the regenerated cellulose membrane of the AF4 system, resulting in very low particle recovery. Strong polydispersity in the applied mobile phase, suggested by batch-mode DLS, might also contribute to sample loss by aggregation and deposition inside the instrument.

Behavior in biological media was also tested. The four monodispersed liposomes (F10102, F10103, F20103A, and F20104A) were analyzed by AF4-DLS in the presence of human serum proteins, in order to detect any variation in size distribution and stability in physiological media due to the formation of a bio-corona on the particle surface [21]. To compare data, the same dilution factor and elution method developed for measuring D_h_ in PBS was applied to liposome diluted in PBS containing 10% human serum. Liposomes F10102, F10103, F20103A, and F20104A showed a very similar elugram in the presence of human serum, as compared to PBS alone (Figure 3), suggesting no or minimal interaction with serum proteins.

### 2.3. AUC Confirmed Results Obtained with DLS and AF4-DLS

**Figure 4:**
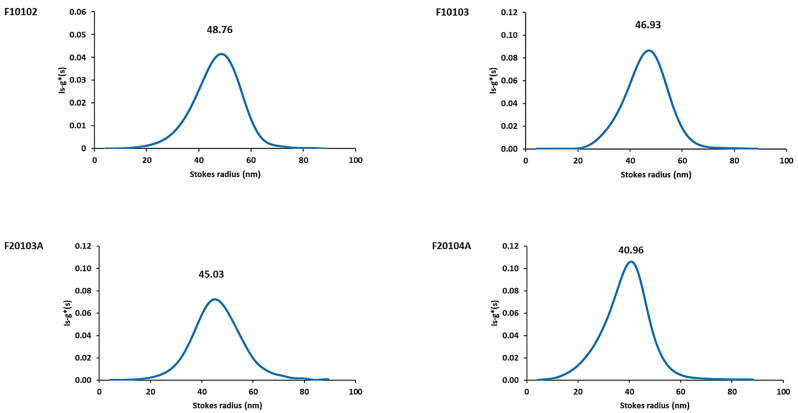
AUC measurement results of liposome size distributions. Stock liposomes were diluted in PBS to a final concentration of 50 μg/mL before proceeding with AUC measurements. PBS was used as control in the reference cell. ls-g*(s) sedimentation coefficient distributions of selected liposomes were calculated from AUC measurements using interference optics and transformed to size distributions. A representative measurement for each of the selected liposomes is reported, with the Stokes radius value expressed in nm.

Liposomes were additionally analyzed by Analytical Ultracentrifugation (AUC), applying the ls-g*(s) model of the Sedfit [22,23,24] to confirm, with an orthogonal technique, their size distribution (expressed as Stokes radius). The interference signal change in the absence of any specific UV-Vis absorption peak was attributed to the liposome sedimentation. Results are reported in Figure 4 and Table 2.

The AUC measurements confirmed the absence of the monodispersed particle population at the expected size range for F50102, F50105, F70101C-NH, and F70101-NH liposomes.

For liposomes F10102, F10103, F20103A, and F20104A, one single sedimentation coefficient (s) distribution peak was detected. According to the results obtained, conversion of s distribution to radius distribution, using the same estimated liposome density, resulted in a Stokes radius (distribution mode) of 48.76 nm for F10102, and 46.93 nm for F10103. Likewise, the Stokes radius of the anionic liposomes, F20103A and F20104A, was calculated to be 45.03 nm and 46.96 nm, respectively, in agreement with the size specifications provided by the manufacturer. The AUC-calculated size distribution showed slightly lower diameters than those obtained with DLS batch-mode and AF4-DLS, probably due to an overestimation of apparent particle density (Table 2).

### 2.4. Bacterial and Endotoxin Contamination

Liposomes were then assessed for potential bacterial contamination, and the results were all negative (data not shown).

They were also tested for endotoxin contamination by end-point and kinetic chromogenic Limulus Amoebocyte Lisate (LAL) assays. Before that, the potential interference of liposomes with the LAL readouts (interference tests at 540 nm and 405 nm) was measured (Supplementary Materials, Figures S2 and S3). The interference data suggest that both assays are suitable for measuring endotoxin in liposomes F10102, F10103, and F20104A, whereas only the end-point assay can be reliably used with liposomes F20103A, F50102, and F50105. Liposomes F70101C-NH and F70101-NH were instead found to interfere very strongly with the readouts of both tests.

Data obtained by both chromogenic LAL assays are summarized in Table 3.

The data show that liposomes F10102, F10103, and F20104A have measurable, although low endotoxin contamination, which could be assessed by both methods giving comparable results. The data obtained by the kinetic test are slightly overestimated (20–35%, as indicated by the recovery rate), while those of the end-point assay are within a 10% variation. F20103A could only be tested with the end-point test and showed a significant endotoxin contamination (around 100 EU/mg). As for the remaining liposomes, F70101C-NH resulted by both methods strongly contaminated, while F70101-NH appeared to have a low contamination. For these liposomes, however, the interference assays showed that neither method is suitable for reliably assessing endotoxin contamination, and therefore these results should not be considered valid. For the cationic liposomes F50102 and F50105, both chromogenic methods were unsuitable, not only because of their interference at the readout wavelength (in the kinetic assay), but also because it was impossible to recover/detect the spiked endotoxin (Table 3 and Supplementary Materials, Figure S2).

Based on these results, three liposomes were selected for further evaluation: F20102, F10103 and F20104A.

### 2.5. Assessment of Liposome Cytotoxicity

The three selected liposomes (F10102, F10103, F20104A) were then tested in vitro for the capacity to directly kill human cells, an important aspect in NP safety assessment.

**Figure 5:**
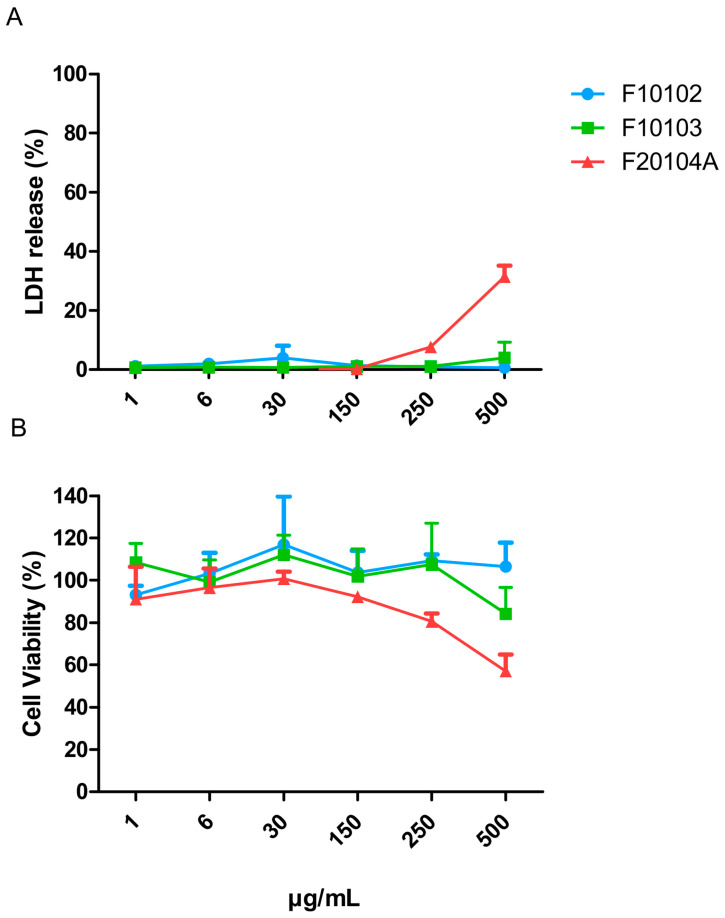
Cell death (% LDH release; **A**) and cell viability (% MTT metabolic transformation; **B**) of Hep G2 cells exposed to liposomes F10102, F10103 and F20104A for 24 h. The error bars represent the SD of three independent experiments. Negative controls (incubation in medium) and positive controls (cell lysis with Triton) were used as benchmarks.

The cell death/membrane damage and the viability/metabolic activity of Hep G2 cells exposed to the liposomes was assessed by the lactate dehydrogenase (LDH) release assay and the 3-(4,5-dimethylthiazol-2-yl)-2,5-diphenyl-2H tetrazolium bromide (MTT) assay, respectively, after 24 h of exposure. Results are presented in Figure 5. No significant cell death (LDH release) was observed upon exposure for F10102 and F10103 at all concentrations tested, even by morphological observations (data not shown), whereas F20104A induced a significant cell death at the highest concentration (500 µg/mL). The percent of metabolically active (living) cells was also decreased significantly by F20204A at the highest concentration, not by the other liposomes tested.

**Figure 6:**
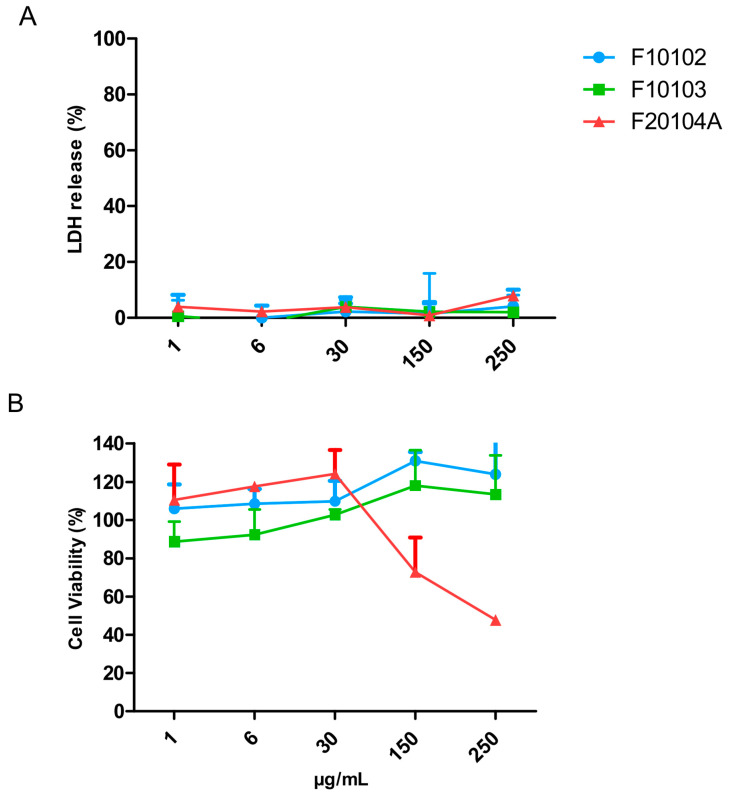
Cell death (% LDH release; **A**) and cell viability (% MTT metabolic transformation; **B**) of PBMC exposed to liposomes F10102, F10103, and F20104A for 24 h. The error bars represent the SD of three independent experiments. Negative controls (cells incubated with medium) and positive controls (cell lysed with Triton) were used as benchmarks.

The potential cytotoxicity of liposomes F10102, F10103, and F20104A was also assessed on human primary blood leukocytes (PBMC), which are a more relevant target compared to the standard assay with Hep G2 cells. Results are presented in Figure 6. No significant cell death (LDH release) was observed upon exposure to F10102, F10103, and F20104A at all concentrations tested, whereas the percent of living cells (MTT assay) was significantly decreased by F20204A at the two highest concentrations tested (150 and 250 µg/mL). F10102 and F10103 did not cause any reduction of cell viability (MTT) at all doses tested.

### 2.6. Effects of Liposomes on Complement Activation

Liposome toxicity was also evaluated in terms of human complement activation.

**Figure 7:**
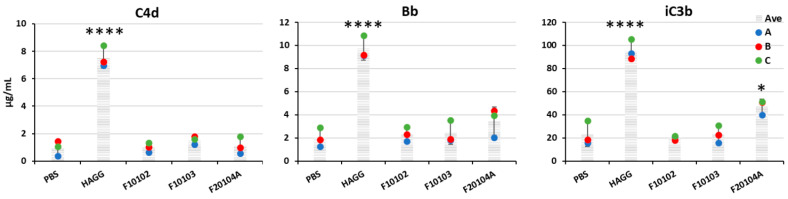
Levels of C4d (left), Bb (center), and iC3b (right) in the plasma of healthy volunteers upon incubation with liposomes. Fresh human plasma from three healthy donors was incubated with liposomes F10102, F10103, or F20104A at 150 µg/mL for 1 h at 37 °C. PBS and heat-aggregated gamma globulins (HAGG, 0.5 mg/mL) were used as negative and positive controls, respectively. Data are shown as individual values (dots of different colors for the three donors) and also as mean (shaded columns) ± SD. * *p* <0.05, **** *p* < 0.0001.

Results in Figure 7 show that, at the highest concentration tested (150 µg/mL), none of the three liposomes induced the generation of C4d in human plasma, suggesting no activation of the classical pathway. Moreover, in terms of Bb generation (suggestive of activation of the alternative pathway), none of the three liposomes resulted in being able to trigger it, while F20104A could induce a significant increase of iC3b, a stable product of the active complement component C3b that is generated by all the complement pathways (classical, alternative, lectin) and leads to the activation of the terminal pathway. No complement activation was observed for any liposome at lower concentrations (data not shown).

### 2.7. Effects of Liposomes on the Production of Inflammation-Related Cytokines by Human Blood Cells

Based on the physical-chemical characterization data, the assessment of bacterial and endotoxin contamination and the cytotoxicity evaluation, only three liposomes (F10102, F10103, F20104A) were selected for the evaluation of immune/inflammatory effects.

Liposomes were used at three concentrations (6, 30, and 150 μg/mL). The concentration of 150 µg/mL corresponded to an endotoxin contamination of 0.12 EU/mL in F10102, 1.26 EU/mL in F10103, and 0.15 EU/mL in F20104A (according to the more reliable end-point test).

It should be remembered that F20104A at 150 µg/mL resulted cytotoxic in terms of reduction of mitochondrial activity in PBMC, suggestive of cytostatic activity, since cell death was not observed. However, no cytotoxic effect was evident at the lower doses of 6 and 30 µg/mL (see Figure 6).

**Figure 8:**
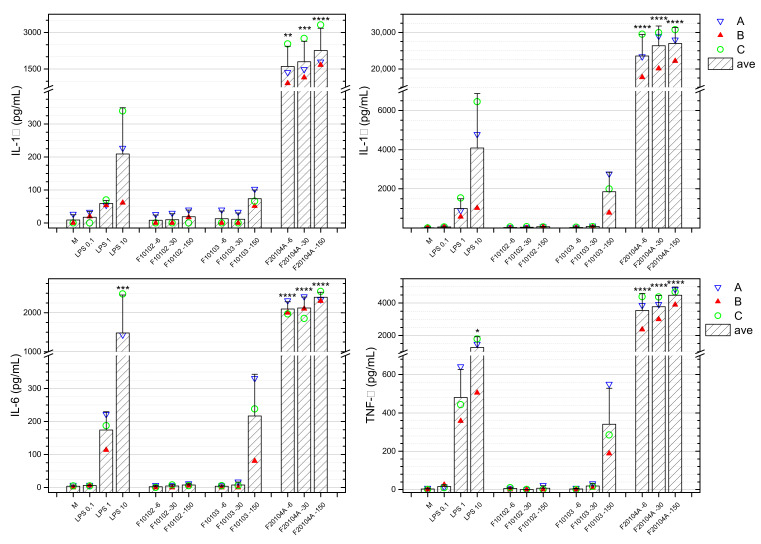
Whole blood from three healthy donors (represented in blue, red, green) was incubated for 24 h with selected liposomes at three concentrations (6, 30, 150 μg/mL). The production of IL-1α, IL-1β, IL-6, and TNF-α data is here reported. M represents the negative control (whole blood exposed to medium only), whereas LPS was used as a positive control at the concentrations of 0.1, 1 and 10 ng/mL. Data are also shown as mean (shaded bars) ± SD. * *p* < 0.05, ** *p* < 0.01, *** *p* < 0.001, **** *p* < 0.0001.

Whole blood from three healthy donors was stimulated with the selected liposomes for 24 h, and the production of a number of soluble cytokines and immune-related factors was assessed by an enzyme-linked immunosorbent assay (ELISA). None of the liposomes could induce significant changes in the basal production of IL-2 and IL-15 (data not shown). Liposome F20104A induced an increased production of IL-1α, IL-1β, IL-4, IL-5, IL-6, IL-10, IL-12p70, IL-13, IL-17, IL-23, IFN-γ, TNF-α, and TNF-β, which were evident at all concentrations tested (Figure 8 and Supplementary Figure S4). Figure 8 reports the results for IL-1α, IL-1β, IL-6, and TNF-α. Data show that liposomes F20104A had a significant effect in stimulating cytokine production in blood cells of all three donors. Conversely, the other liposomes tested had no effect on the cytokine production, with the exception of F10103 at the highest concentration (150 μg/mL). It should be said that at this concentration F10103 showed a level of endotoxin contamination (1.26 EU/mL) slightly above the acceptable level for injectable drugs and is expected to produce some inflammatory effects in human blood cells in vitro. Thus, the increase in the production of inflammatory cytokines may be at least partially attributed to the contaminating endotoxin.

## 3. Discussion

The main goal of this work was to provide a well-structured approach, as well as to share lessons learned, on how to plan a comprehensive characterization of liposome-based nanomaterials for a reliable investigation of their inflammatory effects. It is intended to provide a complete overview to students who are approaching this field for the first time as well as to more skilled developers who might fail in considering some of the aspects of the characterization process.

In this study, we have screened eight liposomes with different charges and compositions, which have been selected as “case-studies”. These are commercially available for drug loading purposes and, according to the producer, have a similar size in the range 100 nm.

As a first, we have performed an adequate physical-chemical characterization, which included the material behavior in complex media. As previously described by Caputo et al. [25], we characterized the particle size and size distribution (PSD) by applying orthogonal techniques as batch-mode DLS, flow-mode DLS-AF4, and AUC. In addition, UV-Vis spectra and ELS have been performed.

The preliminary screening was carried out by batch-mode DLS in order to evaluate the PSD and integrity of monodispersed samples. This technique is usually considered as a fast and simple method but it is not able to reliably analyze polydisperse samples [26,27], since larger particles tend to hide the smaller particle populations [28]. By applying batch-mode DLS to the eight liposomes, only four were found completely monodisperse (F10103, F10102, F20103A, F20104A). For these, batch-mode DLS was also repeated during this time, in order to confirm product stability during the whole study.

DSL data for the three liposomes F50102, F70101-NH, and F70101C-NH upon transformation to number-based distributions are represented by monomodal curves, whereas intensity-based distributions clearly show multimodal distributions. This illustrates the risk of misinterpretation of size distribution data when presented as number-weighted results [29,30].

When DLS is coupled to the AF4 system, in flow-mode, the D_h_ measurement is expected to be more accurate. With this setting, the liposome separation depends on two parameters, i.e., size and surface properties [31,32], however the main advantage is to measure various size fractions separately and avoiding the stronger light scattering effect of larger objects on the size measurement of weak scatterer, small particles. Out of the eight liposomes under investigation, only four were successfully separated by AF4-DLS, corresponding to those resulting as monodispersed particles in the intensity-based distribution of batch-mode DLS.

AF4-DLS was also used to investigate possible variations in size distribution and stability in the presence or absence of serum proteins. This is an important aspect, since upon intravenous administration we expect liposomes to be readily surrounded by serum proteins, which will affect their stability, biodistribution, and bioreactivity [33,34]. In this study, we did not observe changes in the elution behavior in the presence of serum proteins, suggesting that no modification of the liposome surface and size charge occurred.

The selected liposomes were also analyzed by AUC. This is a very powerful technique for the characterization of empty or drug-loaded liposomes [35], although it is not routinely used due to the high instrument cost and the complex data analysis [21]. However, AUC measurements allow the direct generation of mass-based size distributions, due to the linear correlation between liposome concentration and refractive index change [36]. Altogether, we confirmed that Stokes radii of the liposomes were in accordance with the hydrodynamic diameters calculated by batch and flow-mode DLS.

In order to improve the translation of nanoformulations to the clinic, it is important to have the full control of the material under investigation. The unique physical characteristics, such as size and large surface area, make nanomaterials susceptible to undesirable interactions that can hamper their development. As an example, liposome F50105 was certified by the supplier as a monodisperse material, with a size in the range of 100 nm. For this liposome we found, by batch-mode DLS, only one peak (in number- and intensity-based size distribution); however the D_h_ close to 1 µm and the large PDI value clearly indicate that the material had undergone significant aggregation.

The surface charge is another critical parameter, as it can affect cellular uptake, intracellular trafficking pathways, biodistribution, and in vivo fate. Except for F50102, which has a slightly positive charge, all other liposomes were found negatively charged, including F50105, which was described as cationic by the producer.

An important step in the nanomaterial characterization is the evaluation of the possible bacterial and endotoxin contamination (i.e., their sterility and pyrogenicity). Bacterial endotoxin/LPS binds to the Toll-like-receptor 4 (TLR4) on monocytes/macrophages and other cells inducing gene expression and production of many immune/inflammation-related modulators. Among them, IL-1β, TNF-α, IL-6, IL-12, which are involved in inflammatory response [37,38,39,40,41,42,43]. Thus, nanoimmunotoxicity studies that do not consider the accidental presence of endotoxin in particle samples risk attributing to particles the inflammatory effects caused by contaminating endotoxin [10].

In this regard, evaluation of the endotoxin contamination in preclinical NM testing is still not adequately addressed, as reported by Dobrovolskaia and McNeil [44] and as experienced within the H2020 project EUNCL [45]. If, on one hand, testing NM for sterility and bacterial contamination could be easily addressed [46], on the other hand the LAL assay, requested by regulatory authorities for assessing endotoxin contamination in products for human intravenous administration, is not immediately applicable to NM. This is mainly due to the possible interference of NM with the assay readouts and components.

Of the three different variations of the LAL assay (gel-clot, chromogenic, and turbidimetric methods), the chromogenic assay is reported to be the most suitable for measuring endotoxin in NM samples [47,48,49], but its applicability should be tested case-by-case. Since none of the currently available LAL methods is optimal for all types of engineered NM, Dobrovolskaia et al. suggested that at least two LAL formats with different endpoints/readouts should be used [50]. In accordance with this, in our study we have used two chromogenic LAL methods to detect the possible presence of endotoxin in our liposome preparations, i.e., the endpoint and the kinetic assay.

Based on ISO 29701:2010 [51], which provides guidelines for the use of the LAL assay for measuring endotoxin in NP preparation and mentions the possibility that NM can interfere with the assay optical readouts, we have performed UV-Vis analysis and interference tests on all liposomes. To date, not all published studies have addressed this issue, although it should be considered a necessary requirement for reliable measurements. Thus, as recommended by Li et al. [47], we have identified for each liposome the maximal particle concentrations below which no readout interferences is expected.

Another important aspect in running the LAL assay is the use of adequate controls that allow for the identification of other possible interference with the assay components. To this end, it is necessary to “spike” samples with a known amount of endotoxin and assess its recovery. The presence of NM can significantly interfere with the assay components and increase the readout (leading to erroneously recover more endotoxin than added/present) or inhibit it (leading to erroneously recover less endotoxin than added/present). A good recovery rate of the spiked samples has to be in the range of 50–200% of the endotoxin spike [52], although a more restricted range and a more precise endotoxin assessment is preferred when functional immunotoxic/inflammatory evaluation should be performed [53]. Based on our data, the end-point method provided the best spike recovery, therefore values obtained with this method were used to calculate the level of endotoxin contamination of the materials. It must be noted that both the end-point and the kinetic methods were unsuitable for detecting endotoxin contamination in cationic liposomes, as endotoxin in the spike sample could not be recovered, suggesting a strong interference with the assay. This is an important factor to consider when dealing with cationic liposomes, in particular considering their promising applicability as vaccine adjuvants or for gene delivery [8,54,55,56]. The difficulties in detecting potential endotoxin contamination in positively charged liposomes using the current methods suggests that in most cases the experimenters are unaware of the real contamination in their liposome preparations [57]. In the case of cationic liposomes, it would be necessary to adopt different methods to measure endotoxins, such as functional TLR4-based assays [58].

Based on the evaluation of the liposome physical-chemical characteristics, those showing a similar mean diameter size, a narrow size distribution, and limited endotoxin contamination were considered for further in vitro studies of toxicity and inflammatory activation.

For all three liposomes, we selected a concentration of 150 µg/mL as the highest point to use for in vitro functional assays. Based on the LAL assay results, this corresponds to an endotoxin level of 0.12 and 0.15 EU/mL for liposomes F10102 and F20104A, respectively, values which are below the European Pharmacopoeia acceptance value for injectable drugs (0.25 EU/mL) [53]. Liposome F10103 was also included for further experiments, although at 150 μg/mL its endotoxin level (1.26 EU/mL) was above the accepted value. However, at lower concentrations (30 and 6 μg/mL), the endotoxin contamination corresponded to 0.25 and 0.05 EU/mL, respectively, both within the acceptable limit. It should be noted that the accepted values for injectable drugs may not correspond to the concentrations below the activation threshold in vitro, which can be very different and vary depending on the cell type (with monocytes being more sensitive than fibroblasts, as an example), animal species (with humans being more sensitive than mice) and many other factors.

In vitro cytotoxicity testing is an integral part of the procedure for the identification of the hazardous potential of drug candidates in the early phase of drug development. This provides initial insights into the toxic potential of a formulation at low costs and in a short timeframe [31,59]. Safety data for nanoparticulate materials can be obtained by testing according to guidelines published by international standards development organizations [60,61]. Therefore, the three liposome preparations that passed the first two steps (Figure 1) were assessed for direct cytotoxicity in vitro on human hepatocarcinoma Hep G2 cells using a standard protocol [60]. None of the three products showed cytotoxic effects in terms of the number of viable cells (metabolically active cells) and as the number of dead cells (disruption of membrane integrity), even at the highest dose tested (150 µg/mL).

Based on the previous considerations, we tested cytotoxicity also on more relevant cells, i.e., primary cells (rather than tumor cells) and immune cells (rather than epithelial cells). We used PBMC, i.e., the white mononuclear cells that circulate in the human blood, mainly lymphocytes and monocytes. When the cytotoxicity assays were repeated in PBMC, the liposome F20104A resulted to be cytotoxic at the dose of 150 µg/mL, as it causes a reduction of approximately 30% in the metabolic activity. This highlights the fact that, although testing on carcinoma cell lines is meaningful, especially when looking at anti-cancer activity, it is recommended to perform cytotoxicity assessment also on more relevant cells.

It is well known that liposomes can activate the complement system [1,62], complement being the most important and rapid innate/inflammatory defensive system, based on three different pathways (classical, alternative, and lectin pathways) initiated by different kinds of agents and based on a cascade of different soluble components present in plasma, whose activation ends up in the lysis of bacteria and other cells (terminal pathway). Complement is highly conserved throughout evolution, which underlines its efficacy in host defenses. Complement activation by foreign particles may be transient, causing little/no permanent damage to the host, while long-term activation may pose a significant health threat. Therefore, the liposome effects on the human innate immune/inflammatory activation has been first tested in terms of complement activation, using plasma from three different donors. We observed that only the anionic liposome F20104A at the concentration of 150 µg/mL could activate the terminal complement pathway, while none of the three liposomes could significantly induce the generation of C4d (classical pathway) and Bb (alternative pathway). This is in agreement with the previous finding that F20104A at this concentration has significant cytotoxicity for human blood cells.

In addition to direct toxicity for human blood cells and capacity to activate the complement cascade, we eventually tested the capacity of the selected liposomes to induce an inflammatory response. NM could be intentionally engineered to promote an immune/inflammatory response, e.g., by activating cytokine expression, and this property is gaining attention in the vaccine field. In contrast, uncontrolled induction of cytokine response may cause excessive immune stimulation and inflammation with health-threatening consequences [44]. In our study, we selected a panel of cytokines that represent the main response of human blood immune cells. These include potent inflammatory factors (IL-1α, IL-1β, IL-6, IL-12p70, IL-15, IL-17, IL-23, IFN-γ, TNF-α), anti-inflammatory factors, and factors involved in type 2 alternative inflammation (IL-4, IL-5, IL-10, IL-13) and cytokines with activating/homeostatic functions produced by lymphocytes (IL-2, TNF-β). In our study, no production of IL-2 and IL-15 could be observed in any condition (data not shown). Our data show that, at all concentrations tested, the liposome F10102 is unable to activate human blood cells in terms of production of any of these factors. Conversely, F10103 could induce a significant production of IL-1α, IL-1β, IL-6, and TNF-α at the highest concentrations (150 µg/mL), which might be attributed to its significant endotoxin contamination (1.26 EU/mL, i.e., an amount of endotoxin that can elicit monocyte inflammatory activation in vitro).

Eventually, liposome F20104A elicited a significant production of all cytokines (except IL-2 and IL-15) at all concentrations tested. Even excluding the highest concentration (150 μg/mL), which caused cytotoxicity, the effect appeared to be dose-dependent for IL-4, IL-10, IL-12p70, IL-13, and TNF-β, while maximal stimulation was already evident at the lowest liposome concentration in the induction of IL-1α, IL-1β, IL-5, IL-6, IL-17, IL-23, IFN-γ, and TNF-α. This implies a much higher sensitivity to the activation of the genes for the latter factors, which are those more strictly linked to classical inflammation, while the other genes (anti-inflammation and alternative inflammation) appear to need a more potent activation signal. This behavior underlines an important concept, i.e., the fact that a lower particle concentration may have qualitatively different functional effects compared to a higher concentration. In the case of F20104A, the concentration of 6 μg/mL elicited a maximal production of the classical inflammatory cytokines IL-1β, IL-6, IL-17, and TNF-α, while completely unable to trigger the production of anti-inflammatory and alternative cytokines such as IL-10, IL-4, and IL-13. This implies that at this concentration the F20104A liposomes induce a classical inflammatory response (which is also tumoricidal) without feed-back mechanisms. At higher concentrations (30 μg/mL), liposomes F20104A induce the same amount of the classical inflammatory factors but also high amounts of the anti-inflammatory and alternative cytokines, implying that classical inflammation is inhibited and alternative reactions are active. This suggests that the dose, in addition to the particle type, determines the type of biological reaction and whether it may be beneficial or detrimental. Thus, NM toxicity in vitro cannot be reliably evaluated without testing a number of different factors involved in inflammation and its control and without testing several concentrations of the NM.

The reasons for the powerful activation capacity of F20104A were not a matter of investigation in this study, but we can propose some hypotheses and additional controls. One possibility is that the effect can be due to its lipid composition, as F20104A is the only liposome in our study that is composed by HSPC/CHOL 50:50, thus it can be the presence of HSPC (vs. DPPC and DSPC in F10102 and F10103) that triggers cell activation. In this case, the activation observed could be directly attributed to the liposomes. On the other hand, F20104A also presents an ammonium gradient (i.e., an internal ammonium concentration higher than in the outside environment, to allow for rapid and efficient drug loading). Such an ammonium gradient may have loaded the liposomes with bioactive molecules present in the microenvironment (anticoagulated blood) that, upon liposome uptake by blood leukocytes, could have caused a strong cell activation. In this case, the activation would not be attributable to the liposomes themselves but to the unknown cargo acquired during the in vitro experiment. Unfortunately, other liposomes with the same ammonium gradient, F20103A, were highly contaminated by endotoxins and therefore were not tested in the WBA. In any case, this observation suggests that great care should be used when interpreting nanotoxicity data in vitro, in order to distinguish between bona fide NM effects and effects due to the experimental conditions or acquired contaminants.

Thus, excluding F20104A because we do not yet know the reasons of its high immune/inflammatory activation capacity, out of the eight liposomes tested, only F10102 and F10103 seem to be promising candidates for targeted drug delivery.

Our “case-studies” highlight the importance in selecting the most suitable vehicle to be used in clinical formulation, even before evaluating the cargo-loaded liposomes.

Data reported are based on a small cohort of donors, as the scope of the work was focused on providing a model example of a correct approach for a proper characterization strategy. However, differences might be observed among donors’ responses, highlighting the inter-individual variability of the immune responses, and the necessity to move towards more personalized nanomedicine [14].

Careful monitoring of cytokines is necessary to assess the impact of nanomedicines that influence the immune response, particularly during the early phase of product development. As multiple cytokines may be differentially affected, techniques that measure panels of cytokines are most valuable [63].

In conclusion, this work provides a well-structured approach, based on lessons learned, on how to plan a good characterization of liposome-based material, including the critical issue of how to examine and critically evaluate their immune-related effects in terms of induction/modulation of an inflammatory response.

Although the reader might find these results obvious, a large proportion of the selected, commercially available carriers failed to pass the first homogeneity tests, and further products were found to be cytotoxic or interact with the immune system in an undesired way. Similarly, the experience acquired within the recent EUNCL H2020 project shows that in most cases, in the initial phase of the study, product developers are not adequately looking at critical factors such as stability, homogeneity, bacterial, and endotoxin contamination or do not address appropriately the toxicity of the material using reliable and predictive in vitro cell models. As a consequence, product development may fail, after years of investment and research, mainly due to an inappropriate product evaluation at its early stage.

## 4. Conclusions

Despite the increasing number of therapeutic lipid-based NM that the pharma industry and the academic community are translating from benchtop to clinical use, the EUNCL experience showed that, in nearly 40% of cases, the candidate nanomedicines fail at an early stage of the characterization process.

This work provides a rational procedure for assessing the safety of liposomes at the initial stage of their pre-clinical development, which implies the realistic and valid evaluation of their capacity to elicit an inflammatory response in human leukocytes in conditions that eliminate the interference of biologically active contaminants. This underlines the need for an accurate characterization of liposomes at multiple levels, and the use of reliable in vitro methods, in order to provide a realistic assessment of liposome-induced human inflammatory responses, as a fundamental requirement in nanosafety regulations.

## 5. Materials and Methods

### 5.1. Selection of Liposomes

Liposomes with different surface functionalization were purchased from FormuMax Scientific, Inc. (Palo Alto, CA, USA). The main features, as provided by the producer, are summarized in Table 1.

### 5.2. Physical-Chemical Characterization

UV-Vis absorbance spectra were collected using an Evolution 350 UV-Vis spectrometer equipped with the Thermo INSIGHT™2 software (ThermoFisher Scientific, Waltham, MA, USA).

Batch-mode measurements of the particle size distribution (PSD) and polydispersity index (PDI) of liposomes were performed by Dynamic Light Scattering (DLS), using a Zetasizer Nano-ZS (Malvern Instruments Ltd.; Malvern, UK), and data were analyzed by Zetasizer software version 7.12 (Malvern Instruments Ltd.). The same instrument was also used to assess the zeta-potential of liposomes (Electrophoretic Light Scattering—ELS).

The hydrodynamic diameter was measured by applying the Flow-field-flow fractionation (AF4) system (AF2000; Postnova Analytics, Landsberg am Lech, Germany), coupled with a Zetasizer Nano-ZS (Malvern Instruments Ltd.) detector, to determine the size and size distribution of tested liposomes with an orthogonal and more accurate technique. Samples were also prepared in 10% heat-inactivated human AB serum (cat. H4522, lot SLBW8071; Sigma-Aldrich, Inc., St Louis, MO, USA) to check for the effect of serum proteins on liposome size distribution. The AF4-DLS hydrodynamic diameter was calculated from the Z-average values averaged at the time range of half-height peak width of the UV-Vis signal [64,65]. Data were processed by AF200 Control software version 1.1.1.26 (Postnova Analytics) and Zetasizer software version 7.12.

A Beckman Coulter ProteomeLabTM XL-I analytical ultracentrifuge (AUC) (Beckman Coulter Inc., Brea, CA, USA) was used for measuring the Stokes radius of four selected liposomes (F10103, F10102, F20103A and F20104A). The ls-g*(s) model of the Sedfit software was applied to fit experimental data (using linear grid in the 1–500 S range) and to calculate sedimentation coefficient distributions, which were transformed into mass-based size distributions using the “transform s distribution to r distribution” option of Sedfit.

For all these techniques, technical details are reported in the Supplementary information.

### 5.3. Bacterial and Endotoxin Contamination

Selected liposomes were analyzed for potential bacterial and endotoxin contamination.

#### 5.3.1. Assessment of Bacterial Contamination

Liposomes were assessed for potential contamination with viable bacteria using the Luria-Bertani (LB)-Agar test dishes [46]. Several liposome dilutions (0, 1:100, 1:1000 from the stock solution, in sterile LB medium) were plated on duplicate LB-Agar test plates, using as controls LB medium alone and *E. coli* O111:B4 cells. After incubation at 37 °C for 4–72 h, the number of CFU (colony-forming units) was counted.

#### 5.3.2. Assessment of Endotoxin Contamination

The endotoxin contamination was measured using two Limulus amoebocyte lysate (LAL) chromogenic methods, the kinetic assay and the end-point assay modified as described by Li et al. [47] For both methods, a test was run for assessing potential interference of liposomes with the assay readouts (pNA + diazo reagent at 540 nm for the end-point test, and pNA at 405 nm for the kinetic assay) (Supplementary Figure S2). Sample concentrations that were negative in the interference test were used.

For both methods, the glucashield^®^ Reconstitution Buffer (cat. CG1500; Associates of Cape Cod, Inc., East Falmouth, MA, USA) was used for LAL reconstitution. The standard endotoxin from the *E. coli* O111:B4 strain (cat. EC010, 10 ng/vial; Associates of Cape Code, Inc., East Falmouth, MA, USA) was used to prepare the control standard curve (CSE). All dilutions were made in endotoxin-free water (LAL Reagent Water, cat. WP1001; Associates of Cape Code, Inc., East Falmouth, MA, USA).

Standard endotoxin (0.5 EU/mL) was “spiked” in the liposome samples to detect possible liposome interference with the assay. The Pyros Kinetix^®^ Flex Tube Reader (Associates of Cape Code, Inc.) was used to acquire sample readouts at 405 nm, according to the manufacturer’s instruction. The raw data were analyzed with the Pyros^®^ EQS software. For the end-point assay, the readouts were measured at 540 nm with an EnSpire^®^ Multimode plate reader (Perkin Elmer, Waltham, MA, USA). Results for each individual sample were considered valid only if the correlation coefficient of the calibration curve was ≥0.98, and if the recovery rate of spiked controls was within 50% and 200% according to US FDA guidelines [47,66,67]. Assay sensitivity was 0.005 EU/mL and 0.001 EU/mL for the end-point and kinetic methods, respectively.

### 5.4. Cytotoxicity Evaluation

#### 5.4.1. Hep G2 Cells

The in vitro cytotoxicity study of the selected liposomes was evaluated with the colorimetric LDH and MTT assays on Hep G2 cells for 24 h exposure according to the EU-NCL-GTA02 SOPs [68]. The release of the cytosolic enzyme lactase dehydrogenase (LDH) correlates with membrane disruption/cell death, whereas the cleavage of the chromogenic substrate MTT correlates with the cell metabolic activity and therefore with the number of living cells.

Cells of the human hepatocellular carcinoma line Hep G2 were plated in 96-well cell culture plates (Corning Inc., Corning, NY, USA) at a density of 5 × 10^4^ cells/well and allowed to adhere for 24 h, then exposed to the liposomes (1 to 500 μg/mL) for 24 h. Medium control and a positive control (Triton 0.1%) were included in each assay. At the end of the exposure time, 50 µL of supernatant was transferred in a new plate for measuring the release of LDH, which was performed with the BioVision LDH-cytotoxicity colorimetric kit (cat. K311, BioVision, Inc., Milpitas, CA, USA) according to the manufacturer’s instruction. Cell viability was evaluated using MTT [3-(4,5-dimethylthiazol-2-yl)-2,5-diphenyl-2H tetrazolium bromide] (Sigma-Aldrich, Inc.) added to the cells in fresh complete culture medium at a final concentration of 250 μg/mL. After 4 h of incubation at 37 °C the supernatant was removed, the precipitated formazan crystals (indicative of mitochondrial metabolic activity, i.e., presence of viable cells) were dissolved in 200 µL DMSO (Sigma-Aldrich, Inc.) followed by 50 µL of glycin buffer (0.1 M glycine with 0.1 M NaCl). The absorbance was quantitated at 490 nm and 570 nm for LDH and MTT assay, respectively, by the EnSpire^®^ Multimode plate reader (Perkin Elmer) using a reference wavelength of 680 nm. Data are expressed as percent of total LDH release and as percent of mitochondrial activity, and reported as mean ± SD. Three independent experiments were performed in triplicates.

#### 5.4.2. PBMC

In addition to the death/viability assay performed on Hep G2 cells along the EU-NCL-GTA02 SOPs, the potential toxicity of liposomes was evaluated also on human peripheral blood mononuclear cells (PBMC). Approximately 20 mL of peripheral blood was withdrawn by venipuncture from three healthy human donors at the medical service of the Joint Research Centre (JRC), following informed consent and in compliance with the Human Tissue Act (2004) and the Human Tissue Authority Code of Practice 1 [69]. The blood was collected in sterile 9 mL K2 EDTA vacuettes (cat. 455045; Greiner Bio-One GmbH, Kremsmünster, Austria) and diluted 1:1 in PBS. Then, the diluted blood was carefully layered on Ficoll-Paque Plus (GE Healthcare, Chicago, IL, USA) and the mixture was centrifuged at 400× *g* for 40 min. The mononuclear cell layer at the interface between Ficoll and plasma was transferred in another tube and washed 3 times with PBS. The cell pellet was resuspended in 5 mL of RPMI-1640 containing 10% heat-inactivated human AB serum (cat. H4522) and Penicillin-Streptomycin (100 U/mL) (cat. 15140148; ThermoFisher Scientific). PBMC were seeded at 1 × 10^5^ cells/well was seeded in 96-well cell culture plates (Corning, Inc., New York, NY, USA) and immediately exposed to the liposomes (1 to 250 μg/mL) for 24 h. Medium control and positive control (Triton 0.1%) were included in the study. The LDH and MTT assays were performed as described in Section 5.4.1.

### 5.5. Complement Activation Assays

The effect of liposomes on complement activation was measured in plasma samples collected in sterile sodium citrate 3.8% (Vacuette^®^ tubes, cat. 454387, Greiner Bio-One GmbH) from heathy human donors, as described in 2.4.2. Liposomes were prepared in sterile PBS at a concentration of 150 µg/mL and mixed 1:1 *v*/*v* with plasma. PBS and 0.5 mg/mL heat-aggregated gamma globulins (HAGG; cat. A114, Quidel, Santa Clara, CA, USA) were included as negative and positive controls, respectively. Samples were incubated in a final volume of 100 µL for 1 h at 37 °C under orbital shaking at 300 rpm. Then, 10 µL of EDTA 200 mM pH 8.0 were added to block the reaction. The generation of the complement cascade cleavage products iC3b, C4d, and Bb was tested with commercial Enzyme-linked Immunosorbent Assay (ELISA) kits (cat. A006, A008 and A027; Quidel), according to the manufacturer’s instructions after appropriate dilution in specimen diluent (1:100 for C4d, 1:70 for iC3b and 1:10 for Bb). The presence of C4d and iC3b was measured with an EnSpire^®^ Multimode plate reader (Perkin Elmer) at 405 nm, whereas Bb was read at 450 nm. Three independent assays have been performed with plasma from different donors, and a technical replica was run in the ELISA. CV <20% among duplicates was considered acceptable.

### 5.6. Activation of Human Blood Leukocytes

The inflammatory effect of liposomes on human blood leukocytes was assessed by using a whole blood assay (WBA) [70,71].

Approximately 20 mL of peripheral blood was withdrawn by venipuncture from healthy human donors at the medical service of the Joint Research Centre (JRC) as described in 5.4.2. After having discarded the first 6 mL, blood was collected in sterile 9 mL K2 EDTA vacuettes (cat. 455045; Greiner Bio-One GmbH). The WBA was carried out as described by Li et al. [70] A volume of 250 µL of anticoagulated blood was distributed in 1.5 mL Eppendorf tubes. RPMI-1640 culture medium (cat. R8758; Sigma-Aldrich, Inc.) was added to a final volume of 1 mL. Culture medium was used as negative control, while medium containing endotoxin/lipopolysaccharide (LPS from *E. coli* O111:B4, cat. 00–4976; Invitrogen, Thermo-Fisher Scientific) at 0.1, 1, and 10 ng/mL final concentration was used as a positive control. Liposomes were included at the final concentrations of 6, 30, and 150 μg/mL. Tubes were capped and incubated at 37 °C for 24 h. Then, 100 μL of 5% Triton X-100 (cat. 93443; Sigma-Aldrich, Inc.) were added to each tube, samples were centrifuged at 3000× *g* for 10 min at 4 °C and supernatants were stored at −80 °C in small aliquots until test. Three independent experiments were performed.

Samples were thawed on ice and tested for the presence of cytokines using commercial ELISA-based microarrays that simultaneously measure multiple proteins in a single sample aliquot. Multiplex Q-Plex Human Cytokines High Sensitivity Screen (15-plex) from Quansys Biosciences (Logan, UT, USA) was used for assessing the production of IL-1α, IL-1β, IL-2, IL-4, IL-5, IL-6, IL-10, IL-12p70, IL-13, IL-15, IL-17, IL-23, IFN-γ, TNF-α, and TNF-β. Samples were run according to the manufacturer’s instructions. An internal calibrator was used. Cytokines were analyzed with the Q-plex^TM^ Image using the Q-view^TM^ software from Quansys Biosciences. For each cytokine, assay ranges and LOD were provided by the manufacturer.

### 5.7. Statistical Analysis

The GraphPad Prism version 6.01 for Windows (GraphPad Software, La Jolla, CA, USA) was employed for statistical data analysis. Data are reported as mean results of three donors ± SD. Significant differences between treatments were analyzed by ANOVA. A *p* value < 0.05 (95% confidence interval) was considered statistically significant.

## Figures and Tables

**Table 1:** List of liposomes used in the study.

Liposome Description	Lipid Composition	Bulk Buffer Solution	Catalogue nr.	Lipid Stock Concentration (mg/mL)
Plain DPPC/CHOL liposomes	DPPC:CHOL (55:55 mol/mol)	10% sucrose, 20 mM HEPES, <10% ethanol, pH 7.4	F10102	34.6
Plain DSPC/CHOL liposomes	DSPC:CHOL (55:55 mol/mol)	10% sucrose, 20 mM HEPES, pH 7.4	F10103	36.5
DSPC/CHOL liposomes with ammonium sulfate gradient	DSPC:CHOL (55:55 mol/mol)	10% sucrose, 20 mM NaPO_4_, pH 6.5	F20103A	42.2
HSPC/CHOL liposomes with ammonium sulfate gradient	HSPC:CHOL (55:55 mol/mol)	10% sucrose, 20 mM NaPO_4_, pH 6.5	F20104A	36.3
DOTAP/DOPE cationic liposomes	DOTAP:DOPE (50:50 mol/mol)	Nuclease-free water, 10% ethanol, pH 5.5	F50102	3.6
DC-CHOL/DOPE cationic liposomes	DC-CHOL:DOPE (30:70 mol/mol)	Nuclease-free water, pH 5.5	F50105	3.4
Clophosome—high potency clodronate liposomes (neutral)	PC:CHOL (* mol/mol)	PBS (0.9% NaCl, 20 mM NaPO_4_), pH 7.5	F70101C-NH	20.0
Control empty liposomes for Clophosome—high potency (neutral)	PC:CHOL (* mol/mol)	PBS (0.9% NaCl, 20 mM NaPO_4_), pH 7.4	F70101-NH	20.0

CHOL, cholesterol; DC-CHOL, 3b-(*N*-(*N*′,*N*′-dimethylaminoethane)-carbamoyl) cholesterol hydrochloride; DOPE, 1,2-dioleoyl-sn-glycero-3-phosphoethanolamine; DOTAP, 1,2-dioleoyl-3-timethylamonium-propane; DPPC, 1,2-dipalmitoyl-sn-glycero-3-phosphocholine; DSPC, 1,2-distearoyl-sn-glycero-3-phosphocholine; HSPC, hydrogenated phosphatidylcholine; PC, phosphatidylcholine; PBS, Phosphate Buffer Saline. (* mol/mol), not provided by the supplier and declared as proprietary information. Please note that Clophosome contains 20 mg/mL of clodronate disodium.

**Table 2:** Hydrodynamic diameter was measured by batch-mode DLS (intensity-based mean of the most prominent peak) (D_h_) and in flow mode by AF4-DLS (Z-average at peak maximum) (D_h_ *). The Stokes diameter (D_S_) was measured by analytical ultracentrifugation. Zeta-potential and mobility were also determined by ELS. Values are reported as an average of three measurements. SD is reported in brackets. ND: not determined. Monodisperse liposomes in the nano-size range are highlighted in bold.

Liposome	Diameter (nm)			PDI	Zeta-Potential (mV)	Mobility (µm cm/Vs)
	D_h_	D_h_ *	D_S_			
**F10102**	**98.9** **(±26.2)**	**92.2** **(±8.8)**	**96.5**	**0.05**	**−5.90** **(±1.92)**	**−0.40** **(±0.14)**
**F10103**	**104.2** **(±28.0)**	**93.4** **(±10.0)**	**93.9**	**0.06**	**−11.90** **(±2.20)**	**−0.90** **(±0.16)**
**F20103A**	**115.1** **(±31.0)**	**105.6** **(±12.2)**	**90.1**	**0.06**	**−16.60** **(±6.35)**	**−1.20** **(±0.07)**
**F20104A**	**103.7** **(±27.4)**	**96.4** **(±9.8)**	**93.9**	**0.05**	**−11.80** **(±2.09)**	**−0.80** **(±0.15)**
F50102	98.2 (±50.1)	ND	ND	0.36	+4.10 (±0.39)	+0.30 (±0.03)
F50105	929.2 (±119.3)	ND	ND	0.49	−8.10 (±3.40)	−0.60 (±0.26)
F70101C-NH	552.3 (±79.1)	ND	ND	0.59	−7.50 (±0.01)	−0.50 (±0.01)
F70101-NH	1154.4 (±334.2)	ND	ND	0.54	−7.20 (±0.72)	−0.50 (±0.05)

**Table 3:** Endotoxin contamination of liposomes measured by two chromogenic LAL tests. Endotoxin contamination levels are expressed in EU/mg liposomes. In italics, endotoxin evaluations hampered by the significant interference of liposomes with the assays’ readouts (see Figure S2). NM, not measurable. Only three liposomes (bold) were selected for the functional experiments, which are highlighted in bold.

Liposomes	Kinetic LAL Test (OD _405 nm_)		End-Point LAL Test (OD _540 nm_)	
	Recovery Rate (%)	Endotoxin (EU/mg)	Recovery Rate (%)	Endotoxin (EU/mg)
**F10102**	**121**	**1.2**	**99**	**0.8**
**F10103**	**135**	**14.5**	**93**	**8.4**
F20103A	NM	NM	112	100.6
**F20104A**	**131**	**0.7**	**110**	**1.0**
F50102	NM	NM	NM	NM
F50105	NM	NM	NM	NM
F70101C-NH	111	660.0	107	273.3
F70101-NH	97	4.7	87	6.0

## Data Availability

All data related to the study have been presented. They are available at the JRC catalogue: http://data.jrc.ec.europa.eu.

## Supplementary Materials

The following are available online at https://www.mdpi.com/1422-0067/22/2/820/s1.

## Abbreviations

AF4	Asymmetrical Flow-field-flow-fractionation
ANOVA	Analysis of variance
AUC	Analytical Ultracentrifuge
CHOL	Cholesterol
DC-CHOL	3b-(*N*-(*N*′,*N*′-dimethylaminoethane)-carbamoyl) cholesterol hydrochloride
DLS	Dynamic Light scattering
DOPE	1,2-dioleoyl-sn-glycero-3-phosphoethanolamine
DOTAP	1,2-dioleoyl-3-timethylamonium-propane
DPPC	1,2-dipalmitoyl-sn-glycero-3-phosphocholine
DSPC	1,2-distearoyl-sn-glycero-3-phosphocholine
ELISA	Enzyme-linked immunosorbent assay
ELS	Electrophoretic Light Scattering
EMA	European Medicinal Agency
EUNCL	European Nanomedicine Characterization Laboratories
FDA	Food and Drug Administration
Hep G2	Human Hepatocarcinoma G2 cell line
HSPC	Hydrogenated phosphatidylcholine
ICH-S8	Guideline S8 (Immunotoxicology studies for human pharmaceuticals) of the EMA International Council on Harmonization of Technical Requirements for Registration of Pharmaceuticals for Human Use (ICH)
ISO	International Standards Organization
JRC	Joint Research Centre
LAL	Limulus Amoebocyte Lysate
LDH	Lactate dehydrogenase
MTT	3-(4,5-dimethylthiazol-2-yl)-2,5-diphenyl-2H tetrazolium bromide
NM	Nanomaterial
PBMC	Peripheral blood mononuclear cells
PBS	Phosphate Buffer Saline
PC	Phosphatidylcholine
PDI	Polydispersity Index
PSD	Particle Size Distribution
SD	Standard Deviation
TLR4	Toll-like-receptor 4
USNCL	National Cancer Institute-Nanotechnology Characterization Laboratory
