# Supplementary material for: A Step-by-Step Approach to Improve Clinical Translation of Liposome-Based Nanomaterials, a Focus on Innate Immune and Inflammatory Responses
Source: Int J Mol Sci. 2021 Jan 15;22(2):820. doi: 10.3390/ijms22020820 (PMC7830677; doi:10.3390/ijms22020820)
Supplement: Supplementary file 1 [file ijms-22-00820-s001.pdf]

Article

SUPPLEMENTARY MATERIALS

A step-by-step approach to improve clinical translation of liposome-based nanomaterials, a focus on inflammatory response

Giacomo Della Camera [1,2], Dorelia Lipsa [1], Dora Mehn [1], Paola Italiani [2], Diana Boraschi [2] and Sabrina Gioria [1*]

[1] European Commission, Joint Research Centre (JRC), Ispra, Italy; sabrina.gioria@ec.europa.eu

[2] National Research Council (CNR), Institute of Biochemistry and Cell Biology (IBBC), Naples, Italy; paola.italiani@ibbc.cnr.it

* Correspondence: sabrina.gioria@ec.europa.eu; Tel.: +39 0332783584; Fax: +39 0332 785787.

Received: date; Accepted: date; Published: date

1. *Physical-chemical characterization*

UV-Vis absorbance spectra were collected in order to check for absorbance that might interfere with Chromogenic LAL and ELISA tests. All samples were brought to a concentration of 50 μg/mL with ultrapure water, and 0.5 mL of each sample was loaded into a quartz micro cell cuvette (283 QS 1000; Thermo Fisher Scientific). Then, UV-Vis absorbance spectra (λ range = 200-800 nm) were collected in duplicate in scan mode (speed 10000 nm/min) by an Evolution 350 UV-Vis spectrometer equipped with the Thermo INSIGHT™2 software (Thermo Fisher Scientific).

Batch-mode measurements of the particle size distribution (PSD) and polydispersity index (PDI) of liposomes were performed by Dynamic Light Scattering (DLS), using a Zetasizer Nano-ZS (Malvern Instruments, Ltd.). Data were analysed by Zetasizer software version 7.12 (Malvern Instruments, Ltd.). For all liposomes, two dilutions at 25 and 50 μg/mL were prepared in Dulbecco's Phosphate Buffered Saline 9.5 mM phosphate w/o calcium and magnesium pH 7.0-7.6 (PBS, cat. BE17-512F; Lonza, Basel, CH). Then, 0.5 mL of each sample dilution were transferred in a disposable cuvette (cat. ZE0040; BrandTech Scientific, Essex, CT, USA) and measured twice at 25°C, after an equilibration step of 120 sec with an acquisition time of 80 sec. The same instrument was also used to assess the zeta-potential of liposomes (Electrophoretic Light Scattering, ELS). In this case, dilutions at 50 μg/mL were used, and 1 mL volume was loaded in disposable folded capillary cells (cat. DTS1070; Malvern Instruments, Ltd.), and measured for surface charge.

The hydrodynamic diameter was measured by applying the Flow-field-flow fractionation (AF4) system (AF2000; Postnova Analytics), coupled with a Zetasizer Nano-ZS (Malvern Instruments, Ltd.) detector, to determine with an orthogonal and more accurate technique the size and size distribution of tested liposomes. Data were processed by AF200 Control software version 1.1.1.26 (Postnova Analytics) and Zetasizer software version 7.12. For measurements, a regenerated cellulose membrane was selected (cat. Z-A4F-MEM-612; Postnova Analytics) with a 10 kDa cut-off, PBS as eluent, a 350 μm spacer, and a 20 μL loop to inject a volume of 20 μL of diluted liposomes into the AF4 system. Liposomes were diluted in PBS for flow-mode measurements. Samples were also prepared in 10% human serum (cat. H4522, lot SLBW8071; Sigma-Aldrich, Inc.) to check for the effect

of serum proteins on liposome size distribution. During the measurement, the detector flow was kept at 0.5 mL/min, and an optimal size separation of the liposomes was achieved using an injection flow of 0.20 mL/min and a constant cross-flow of degassed buffer solution of 1 mL/min for the first 5 min followed by a power type cross-flow change (1-0.1 mL/min in 60min). Afterwards, the cross-flow was kept constant (0.1 mL/min) for other 10 min. Zetasizer Nano-ZS flow mode measurements were performed in a Hellma Quartz Suprasil 3 mm flow-through cuvette (Hellma & Co. KG, Müllheim, Germany). Both flow and batch-mode measurements were performed at the 173° backscattering angle. Dilutions were selected in order to have a high signal to noise ratio, and to avoid multiple scattering interferences.

A Beckman Coulter ProteomeLabTM XL-I analytical ultracentrifuge (AUC) (Beckman Coulter) was used for measuring the Stokes radius of four selected liposomes (F10103, F10102, F20103A and F20104A), which were diluted to a final concentration of 50 μg/mL in PBS. The volumes of liposome suspension and PBS loaded in the sample cell and reference cell of the centerpiece were both 400 μL. Then, sample holders were placed in an 8-hole rotor, and interference signal was measured at a constant rotation speed of 5000 rpm at 20°C. For the selected liposomes, data were analysed considering a density of 1.04 g/mL. The ls-g*(s) model of the Sedfit software was applied to fit experimental data (using linear grid in the 1-500 S range) and to calculate sedimentation coefficient distributions, which were transformed into mass-based size distributions using the "transform s distribution to r distribution" option of Sedfit.

All reagents, unless indicated were purchased from Sigma-Aldrich, Inc. A 0.22 μm membrane filter system (cat. 566-0020; ThermoFisher Scientific) was used when filtration was applied.

2. Interference test

2.1 Introduction

In 2010, the International Standard Organization (ISO) published the ''Nanotechnologies-Endotoxin test on nanomaterial samples for *in vitro* systems'' [1], and proposed the interference test as a necessary requirement for the reliable detection of endotoxin contamination in nanomaterial preparations by LAL assay. The test consists in evaluating the interference of sample dilutions with LAL assay's components and optical readouts [2, 3].

2.2 Materials and Methods

The interference test was performed as described by Li *et al.* [4] at 405 nm or 540 nm for kinetic or end-point assay, respectively. For both methods, 2-fold dilutions of liposome samples, prepared into LAL reagent water (LRW, WP1001; Associates of Cape Cod, Inc.), were distributed in duplicate in 96-well plates (flat-bottom, cat. CLS3599; Corning, Inc.) in a final volume of 50 μL/well. Then, eight 2-fold dilutions of synthetic 4-nitroaniline (pNA, cat. 185310; Sigma-Aldrich, Inc.) were dispensed, reaching a final volume of 100 μL/well. The 4-nitroaniline is the chemical compound measured in chromogenic LAL assays as readout of endotoxin presence. For the kinetic assay, the optical density of pNA (yellow) in each well was immediately measured with a plate reader at 405 nm. Conversely, for the end-point method an additional step was performed, consisting in the addition of diazo-reagents, and the reading is performed at 540 nm. In detail, 50 μL/well of three different diazo-reagents were added to each well: reagent 1 (cat. 237213; Sigma-Aldrich, Inc.) 0.41 mg/mL $NaNO_2$ in 0.48 N HCl; reagent 2 (cat. 51512; Sigma-Aldrich, Inc.) 3 mg/mL ammonium sulfamate in LRW; and reagent 3 (cat. N9125; Sigma-Aldrich, Inc.) 0.7 mg/mL N-(1-naphthyl)ethylenediaminedihydrochloride in LRW. In this case, the final volume was 250 μL/well. When all diazo-reagents were rapidly added in the right sequence (1, 2, and 3) to wells already containing pNA and liposome dilutions, the pNA turned from yellow to purple, and the optical density of the azo-dye product was measured spectrophotometrically at 540 nm. Optical densities were measured with anEnSpire® Multimode plate reader using the EnSpire® Manager software.

The not-interfering dilutions of each liposome were selected for the subsequent chromogenic LAL assays.

2.3 Results

The possible interference of liposomes with the readouts of the LAL assays at both 405 nm (for the kinetic assay) and 540 nm (for the end-point assay) was investigated.

As first step, the UV-Vis spectra were collected for all liposomes at the concentration of 50 μg/mL. Results are reported in Figure S1, and show no specific peaks of absorption at 405 or 540 nm.

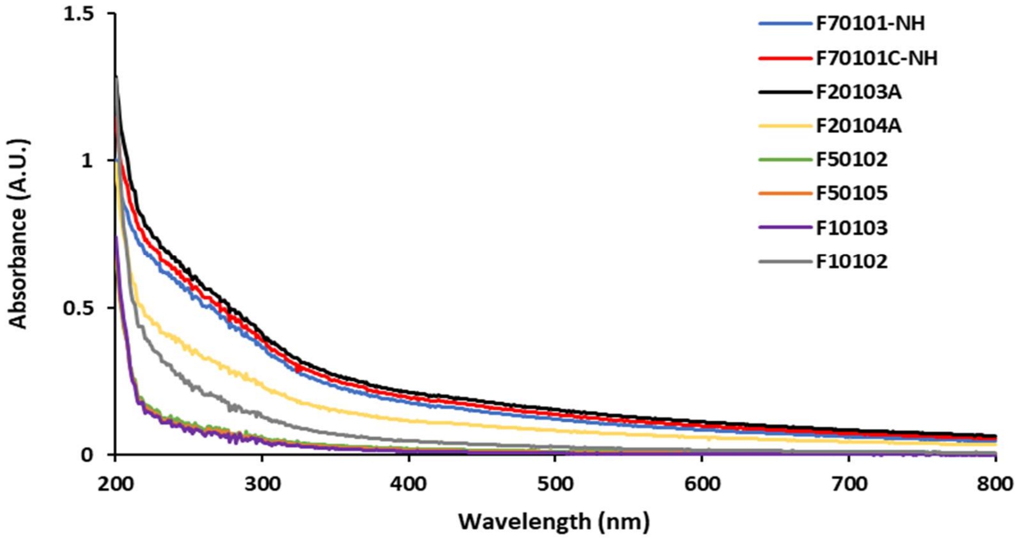

Figure S1. UV-Vis spectra of liposomes. No specific peaks of absorbance are visible at 405 and 540 nm for the tested liposomes. For UV-Vis measurements, samples were used at 50 μg/mL. The averages of two measurements for each liposome are shown.

Then, the interference test was performed as described by Li *et al.* [4].

For the end-point assay, liposomes were tested at concentrations between 62.5 and 250 μg/mL. Results are reported in the Figure S2.

Despite the differences in liposome surface functionalisation, the optical readings at 540 nm of the pNA+azo-dye product were essentially unaffected by the presence of liposomes at all tested concentrations. Exceptions are for F70101C-NH and F70101-NH, which showed interference at 540 nm with an increase of the pNA+azo-dye readout, which was low but detectable even at the lowest concentration tested. Therefore, when running the end-point LAL assay, these two liposomes had to be tested starting from concentration below 62.5 μg/mL.

When assessing the liposome interference with the pNA readout at 405 nm (the wavelength used for the kinetic LAL assay), a concentration range between 31.25 and 250 μg/mL was tested. Results reported in Figure S3 show that none of the liposomes at the concentration of 31.25 μg/mL interfered with the pNA readout at 405 nm, except F50102 and F50105. These cationic liposomes were also tested at a lower concentration (15.625 μg/mL), but still interfered significantly with the readout. Among the other liposomes, F20103A showed a slight interference from 62.5 μg/mL and higher, while F70101C-NH and F70101-NH interfered at 125 and 250 μg/mL (Figure S3). Thus, in the kinetic LAL assay, liposomes F10102, F10103 and F20104A could be tested at every concentration from 250 μg/mL down, F70101C-NH, F70101-NH and F20103A from 31.25 μg/mL, and F50102 and F50105 were tentatively tested at 12.5 μg/mL and below.

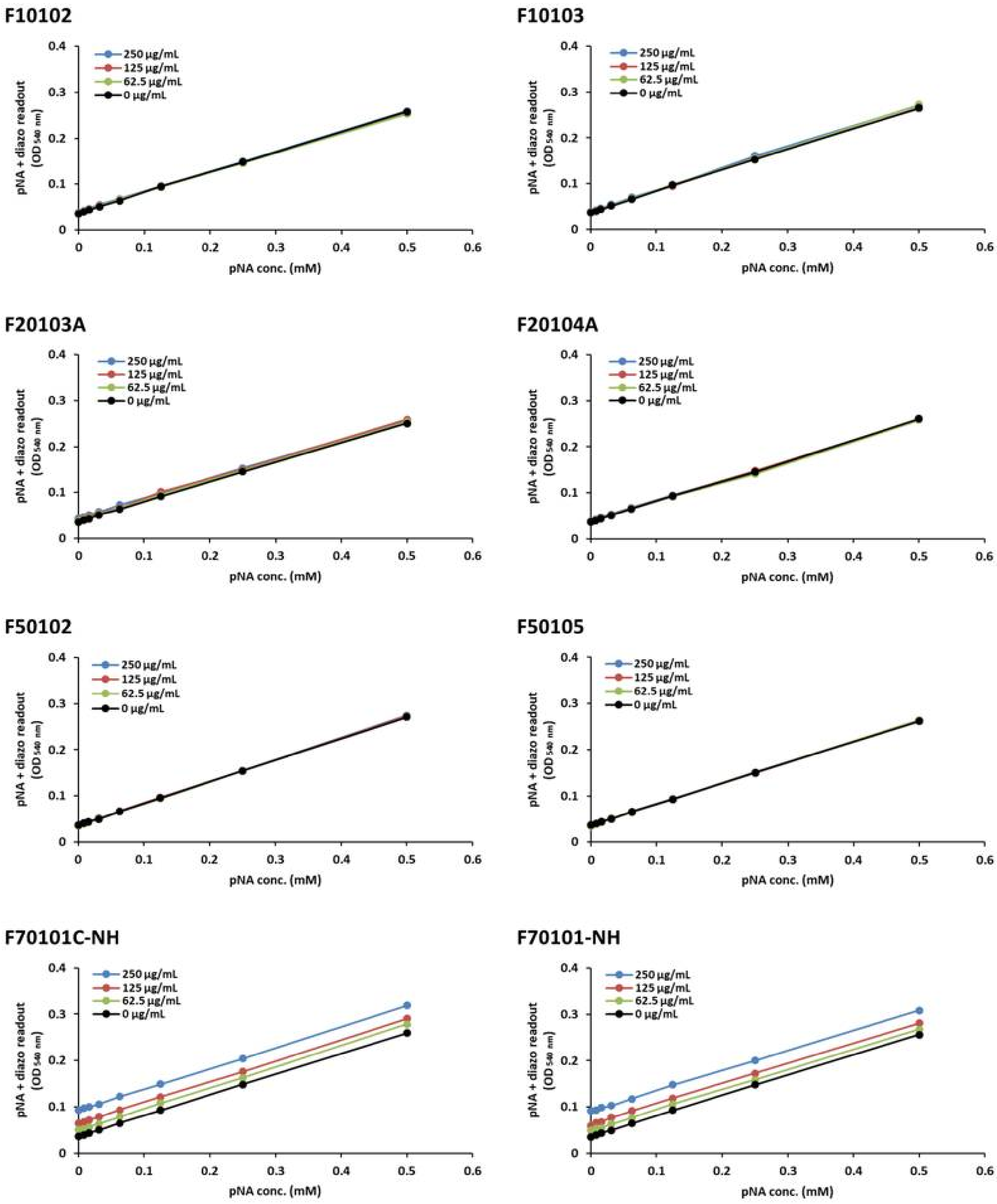

Figure S2. Interference test at 540 nm**.** Liposome interference with the chromogenic LAL assay' readout of pNA+ azo-dye at 540 nm. In the graphs, liposome concentrations are reported with different colours (0 μg/mL represents endotoxin-free water that was used as control). The means of duplicates at the different liposome concentrations are reported.

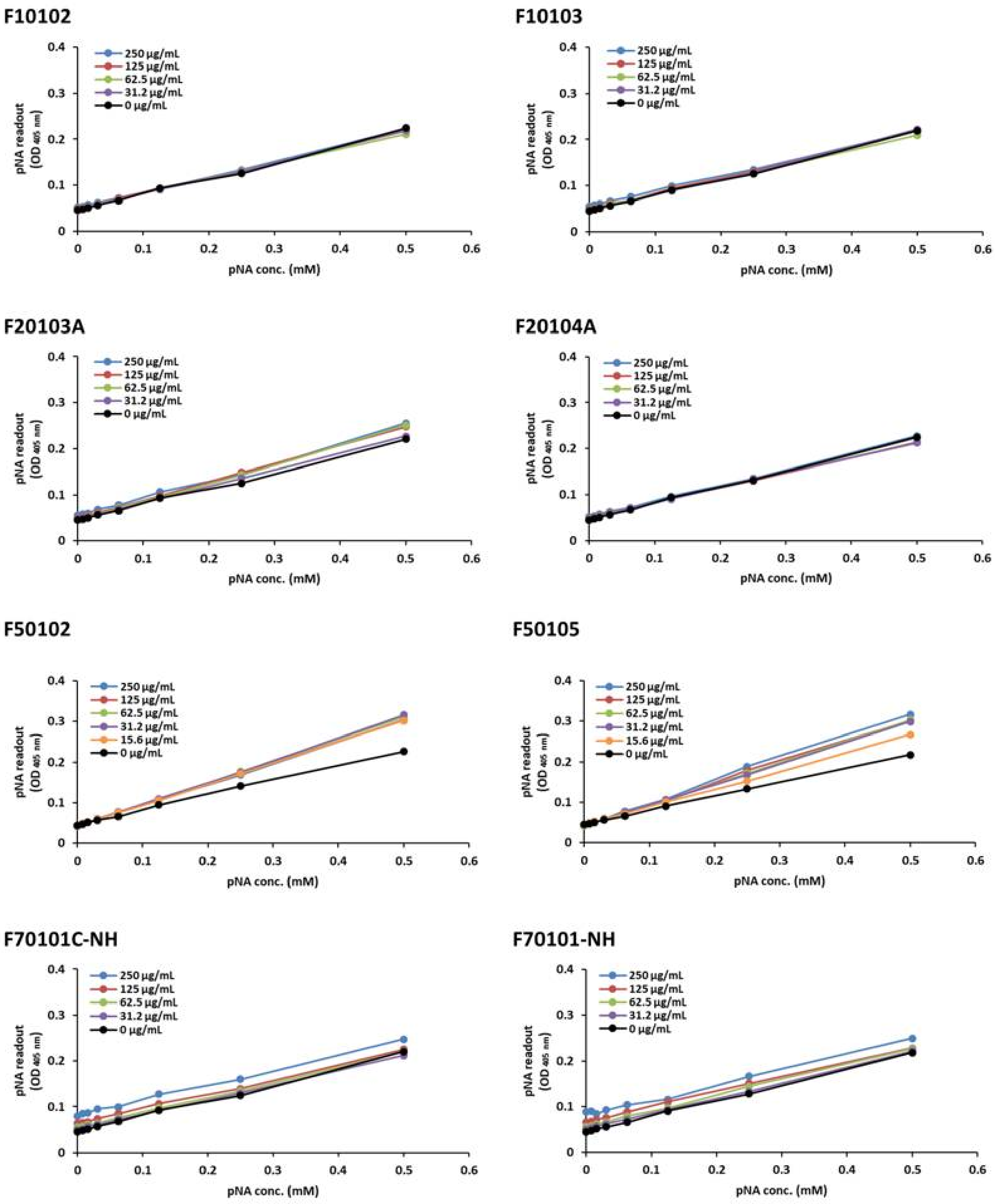

Figure S3. Interference test at 405 nm. Liposome interference with the chromogenic LAL assay' readout of pNA at 405 nm. In the graphs, liposome concentrations are reported with different colours (0 µg/mL represents endotoxin-free water that was used as control). The means of duplicates at the different liposome concentrations are reported.

3. Cytokine production

Whole blood from three healthy donors was stimulated for 24 h with the selected liposomes at three concentrations (6, 30 and 150 µg/mL). The production of a number of soluble cytokines and immune-related factors was assessed by ELISA and presented in Figure S4. IL-4, IL-5, IL-10, IL-12p70, IL-13, IL-17, IL-23, IFN-γ and TNF-β production is reported.

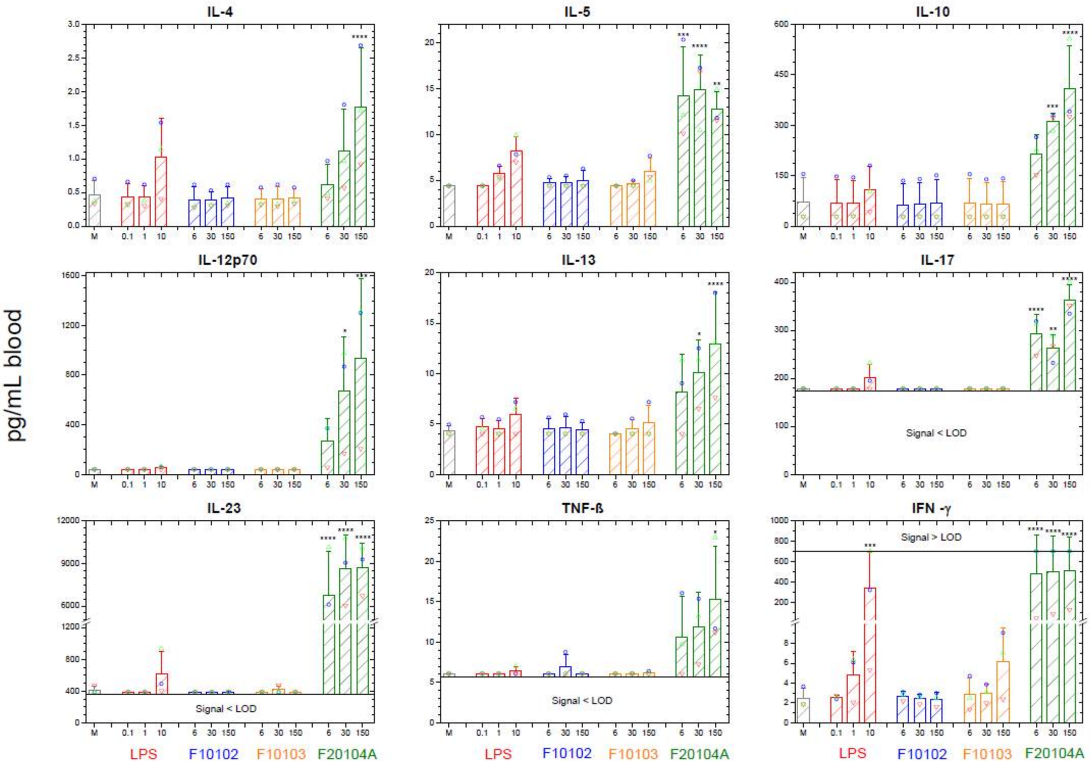

Figure S4. Cytokines secretion in human blood cells in response to liposomes. Whole blood from three healthy donors was stimulated for 24 h with the selected liposomes at three concentrations (6, 30 and 150 μg/mL). The production of a number of soluble cytokines and immune-related factors was assessed by ELISA. IL-4, IL-5, IL-10, IL-12p70, IL-13, IL-17, IL-23, IFN-γ and TNF-β production is reported. IL-2 and IL-15 production was not measurable (below the lower limit of detection, LOD). M represents the negative control (medium), whereas LPS was used as positive control at the concentration of 0.1, 1 and 10 ng/mL. Data are expressed as pg/mL blood and shown as mean of the three donors ± SD. *p <0.05, **p <0.01, ***p <0.001, ****p<0.0001. For IL-17, IL-23, TNF- β and IFN-γ lower or upper LOD are shown.

References

1 'ISO 29701:2010(en). Nanotechnologies — Endotoxin test on nanomaterial samples for in vitro systems — Limulus amebocyte lysate (LAL) test (2010)'. https://www.iso.org/standard/45640.html.

2 Li Y, Boraschi D. Endotoxin contamination: A key element in the interpretation of nanosafety studies. *Nanomedicine* 11(3), 269–87 (2016).

3 Dobrovolskaia MA, Germolec DR, Weaver JL. Evaluation of nanoparticle immunotoxicity. *Nat. Nanotechnol.* 4, 411–414 (2009).

4 Li Y, Italiani P, Casals E, Tran N, Puntes VF, Boraschi D. Optimising the use of commercial LAL assays for the analysis of endotoxin contamination in metal colloids and metal oxide nanoparticles. *Nanotoxicology* 9(4), 462–73 (2015).
